# A Review of an Investigation of the Ultrafast Laser Processing of Brittle and Hard Materials

**DOI:** 10.3390/ma17153657

**Published:** 2024-07-24

**Authors:** Jiecai Feng, Junzhe Wang, Hongfei Liu, Yanning Sun, Xuewen Fu, Shaozheng Ji, Yang Liao, Yingzhong Tian

**Affiliations:** 1 School of Mechatronic Engineering and Automation, Shanghai University, 99 Shangda Road, BaoShan District, Shanghai 200444, China; 2 Ultrafast Electron Microscopy Laboratory, The MOE Key Laboratory of Weak-Light Nonlinear Photonics, School of Physics, Nankai University, Tianjin 300071, China; 3 State Key Laboratory of High Field Laser Physics, Shanghai Institute of Optics and Fine Mechanics, Chinese Academy of Sciences, Shanghai 201800, China

**Keywords:** brittle and hard materials, ultrafast laser processing, mechanism, energy absorption, energy transformation, precise manufacturing

## Abstract

Ultrafast laser technology has moved from ultrafast to ultra-strong due to the development of chirped pulse amplification technology. Ultrafast laser technology, such as femtosecond lasers and picosecond lasers, has quickly become a flexible tool for processing brittle and hard materials and complex micro-components, which are widely used in and developed for medical, aerospace, semiconductor applications and so on. However, the mechanisms of the interaction between an ultrafast laser and brittle and hard materials are still unclear. Meanwhile, the ultrafast laser processing of these materials is still a challenge. Additionally, highly efficient and high-precision manufacturing using ultrafast lasers needs to be developed. This review is focused on the common challenges and current status of the ultrafast laser processing of brittle and hard materials, such as nickel-based superalloys, thermal barrier ceramics, diamond, silicon dioxide, and silicon carbide composites. Firstly, different materials are distinguished according to their bandgap width, thermal conductivity and other characteristics in order to reveal the absorption mechanism of the laser energy during the ultrafast laser processing of brittle and hard materials. Secondly, the mechanism of laser energy transfer and transformation is investigated by analyzing the interaction between the photons and the electrons and ions in laser-induced plasma, as well as the interaction with the continuum of the materials. Thirdly, the relationship between key parameters and ultrafast laser processing quality is discussed. Finally, the methods for achieving highly efficient and high-precision manufacturing of complex three-dimensional micro-components are explored in detail.

## 1. Introduction

The femtosecond laser, with the characteristics of an ultra-short pulse and high instantaneous power, was first developed by E. B. Treacy from the United Aircraft Research Laboratories of the United States in 1969 [1]. Subsequently, femtosecond laser technology progressed from ultrafast to super-strong due to the development of chirped pulse amplification technology [2,3,4,5]. It quickly became a flexible tool used in medical [6,7,8,9], aerospace [10,11] and optics [12,13] fields for machining complex microstructures out of brittle and hard materials such as superalloys [14,15,16,17], thermal barrier ceramics [18,19], diamond [20,21,22,23], silicon dioxide [24] and silicon carbide [25,26,27,28] and so on.

Continuous wavelength lasers and nanosecond lasers mainly utilize the resonance linear absorption of electrons on laser photons to heat the materials [29,30,31]. The processes involve melting, evaporating, vaporizing, and removing the materials: a hot melting process [32]. The interaction time between those lasers and the materials is longer than the thermal diffusion time of the materials. Thus, continuous wavelength lasers and nanosecond lasers are mainly used in the processing of metal and alloy materials due to the large heat-affected zone [33], which greatly limits their application to non-metallic materials [34,35].

**Figure 1:**
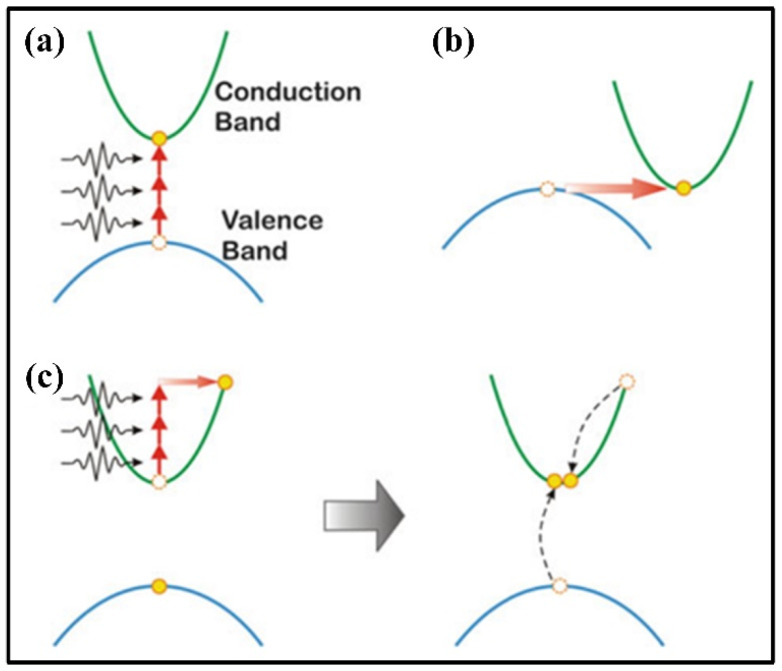
Nonlinear photoionization of femtosecond laser processing: (**a**) multiphoton ionization, (**b**) tunneling ionization, and (**c**) avalanche ionization [43].

However, picosecond and femtosecond lasers would induce high-density plasma around the laser beam focus position in the materials mainly due to multiphoton ionization, photoionization, and avalanche ionization because of the ultrashort pulse duration and extremely high peak intensity of an ultrafast laser [36,37,38]. Thus, the microstructure of the materials around the laser beam focus position is modified as the energy of the femtosecond laser is transferred to the materials via the ionization of the electrons in the materials. The characteristics of femtosecond laser micro–nano processing are mainly in the following three aspects. Firstly, femtosecond lasers can approximately achieve cold processing because of their ultra-short pulse time, low single-pulse energy and extremely small heat-affected zone. Secondly, femtosecond lasers can realize sub-wavelength spatial processing accuracy as the energy of the laser can be absorbed by the materials via nonlinear absorption, such as in the case of multiphoton ionization [39], tunneling photoionization [40], and avalanche ionization [41] (Figure 1). Finally, femtosecond laser micro-machining can be applied to a variety of materials, such as ceramics, diamond, and silica metals, because the breakdown threshold of the materials is dependent on their bandgap energy [42].

Materials processing has been revolutionized by femtosecond lasers with their ultrashort pulse durations and high peak intensity [44,45,46]. Research on femtosecond lasers mainly focuses on the interaction mechanisms between a femtosecond laser and materials, the processing of materials using femtosecond lasers, and applied research [47,48]. Firstly, in the research focused on the interaction mechanisms, the interaction mechanisms of a femtosecond laser with metals, ceramics, and other materials have been investigated. The two-temperature model (TTM) was established to reveal the interaction mechanisms between a femtosecond laser and metals [49]. However, the existing double-temperature model might require further improvement in order to be adapted to non-metallic materials [50,51]. Secondly, in the context of processing materials using a femtosecond laser, the ablation threshold [52,53,54], induced micro–nano structures [55,56,57,58], material densification [59] and local refractive index modification have been investigated. Finally, in applied research, femtosecond laser manufacturing of high-quality film-cooling holes for turbine blades [60,61,62], X-ray lenses for optical devices [63], optical splitting crystals with a synchrotron radiation source [64], and precision surface microstructures for aerospace components [65,66,67,68,69] is widely applied.

However, femtosecond laser processing still faces many challenges. Firstly, the nonlinear absorption mechanisms and the coupling mechanisms of energy transfer and transformation of materials with different physical properties are still unclear [70,71]. Secondly, a cross-scale in situ monitoring system needs to be established [72,73]. Thirdly, data-driven process modeling needs to be established [74]. Finally, integrated software-controlled processing equipment should be developed to meet the requirements of efficient and precise manufacturing in the industry [75,76,77,78].

This review is focused on the common challenges and current status of the ultrafast laser processing of brittle and hard materials, such as nickel-based superalloys, thermal barrier ceramics, diamond, silicon dioxide, and silicon carbide composites. It should be noted that some important brittle and hard materials, such as tool steels and martensitic and carbide steels required to work at high temperatures and cast steels with high carbon contents, could not be adequately discussed in this paper due to length limitations. This paper is organized as follows. Section 2 illustrates the mechanisms of the interaction between an ultrafast laser and brittle and hard materials. Section 3 introduces the ultrafast laser processing of brittle and hard materials. Section 4 reviews the precise manufacturing of ultrafast laser processing. Finally, the present challenges and future prospects are given in Section 5.

## 2. Mechanism of the Interaction between Ultrafast Laser and Brittle and Hard Materials

### 2.1. Absorption of Ultrafast Laser Energy

**Figure 2:**
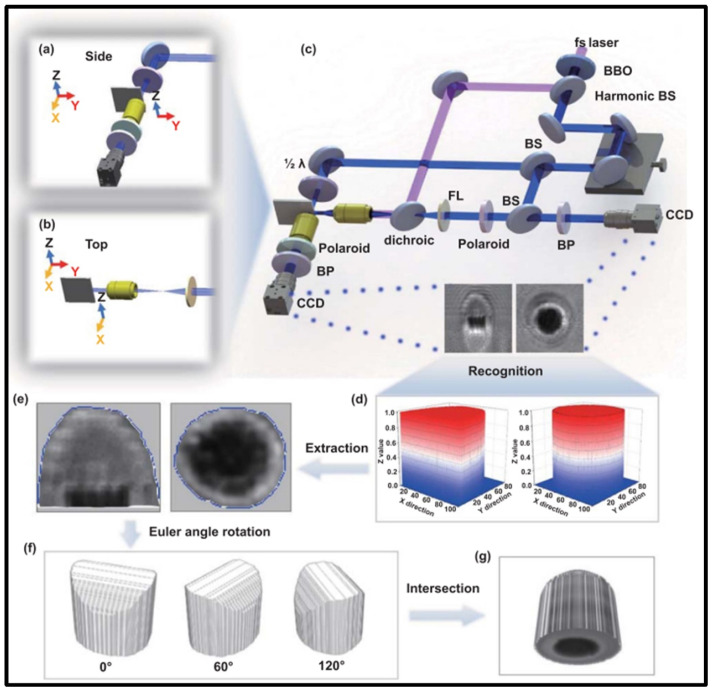
Scheme of the ultrafast quasi-3D imaging method: (**a**) top view, (**b**) side view, in (**c**), ablation and eruption dynamics of a sapphire, (**d**) recognition, (**e**) extraction, (**f**) rotation, and (**g**) intersection [85].

The primary interaction between a femtosecond laser and materials is via the absorption of photons, which excites electrons from a state of equilibrium to some excited states [79]. For semiconductors, electrons on the valence band can be motivated to the conduction band by absorbing a single photon, i.e., linear absorption. However, for wide-bandgap materials, the nonlinear absorption [80] of ultrafast laser energy by materials with different properties involves multiphoton ionization [81,82], tunneling photoionization [83], avalanche ionization, and other physical processes. The absorption mechanisms of materials in relation to the energy of the laser should be tailored to different materials according to their bandgap width, thermal conductivity, and other characteristics. The instantaneous dynamic characteristics of laser-induced plasma and its electrons are key to affecting the energy absorption of materials. The femtosecond laser processing of materials could induce a free electron plasma whose density is contingent on physical phenomena such as single-electron diffusion, multiphoton ionization, and electron–hole radiation recombination [84]. Lian et al. [85] reported that an innovative quasi-3D imaging method could be used to analyze the laser-induced plasma dynamics in femtosecond laser processing, which would provide a more comprehensive understanding and elucidate a variety of complex ultrafast laser processing phenomena (Figure 2).

Based on the dual-temperature model, ablation threshold theory, and liquid phase blasting theory, ultrafast laser dynamics simulations could be carried out to examine the absorption of femtosecond laser energy by materials and experiment with ultrafast laser processing. Spectral detection and three-dimensional ultrafast continuous imaging could be integrated into a scanning electron microscope (SEM) system. An in situ monitoring system with a high spatiotemporal resolution could be established to analyze the evolution mechanisms of plasma and the absorption processes involving laser energy. Combined with molecular dynamics simulations [86,87,88,89], the nonlinear absorption mechanisms of ultrafast laser energy of different physical materials could be clarified, giving a new understanding of the nonlinear absorption mechanisms of ultrafast lasers and lay a theoretical foundation for researching ultrafast laser processing methods.

**Figure 3:**
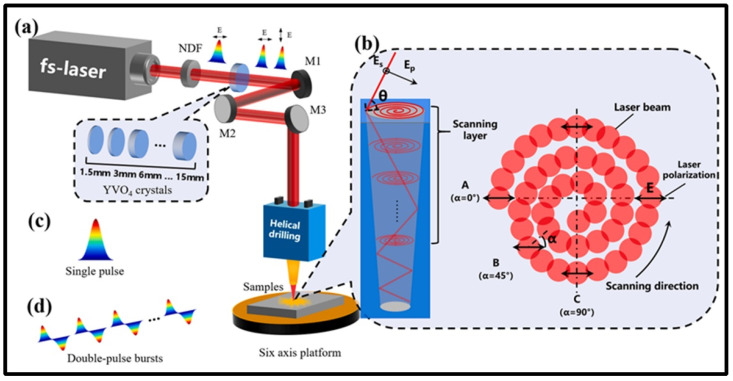
Femtosecond laser processing system: (**a**) schematic diagram, (**b**) parameter changes during micro-hole drilling, (**c**) single-pulse and (**d**) double-pulse bursts [90].

Femtosecond laser processing has attracted attention from researchers involved in the precision machining of superalloys coated with thermal barrier coatings (TBCs), as femtosecond lasers have a short pulse width and high peak intensity. During femtosecond laser irradiation, free electrons are heated instantaneously due to the absorption of photon energy. The process of absorbing photon energy establishes a non-equilibrium state between the electronic system and the lattice system, promoting the rapid coupling of electron phonons and inhibiting thermal diffusion. Qiu et al. [90] invented an orthogonally polarized femtosecond laser to drill Ni-based superalloys with a TBC and produce high-quality film-cooling holes (Figure 3).

**Figure 4:**
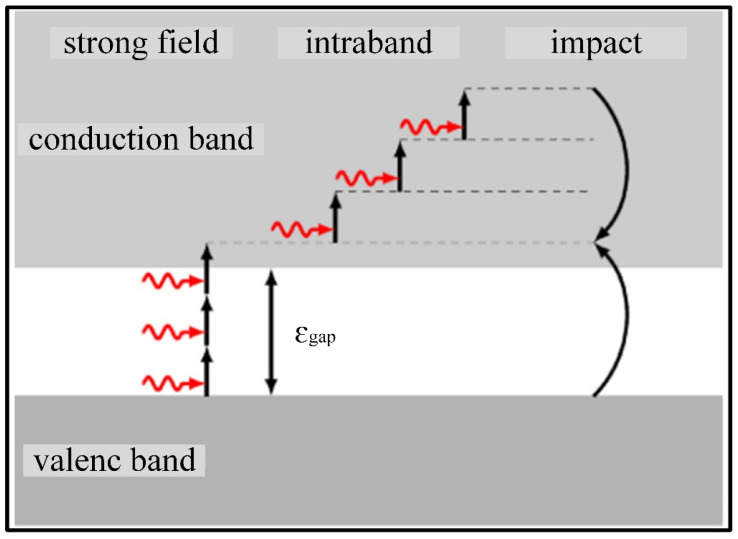
Schematic diagram of electron excitation process in the ultrafast laser process [91].

Diamond is a kind of wide bandgap material. Its free electron density increases sharply under the excitation of ultrashort laser process. The production of free electrons transforms the insulating diamond into a conductive material with metallic optical properties, as shown in Figure 4 [91].

Electrons can move to a higher state by absorbing photons. This transformation phenomenon can be presented using the multi-rate equation (MRE) model. Light attenuation is caused by the strong field absorption and free carrier absorption indicated in the Drude diagram. Smirnov et al. [92] confirmed that two-photon absorption was the main absorption mechanism during the nonlinear absorption of the ultrashort laser processing of diamond, with laser intensities of 0.17–1.7 TW/cm^2^.

Silicon carbide (SiC) is a representative wide-bandgap material used in new-generation semiconductors. Additionally, SiC is also a material with a high hardness, following that of diamond. Thus, the processing of SiC is a challenge [93]. SiC materials mainly absorb laser energy via multiphoton absorption. Material removal during laser ablation processes is mainly carried out via two mechanisms: plasma vaporization and explosion. Compared with nanosecond and picosecond lasers, femtosecond lasers with shorter pulse width and high peak intensity can directly ionize materials with almost no thermal effect. The absorption spectra analysis showed that the SiC colloid solution has strong absorption in the ultraviolet region. The nonlinear absorption characteristic of the SiC nanoparticle colloidal solution contrasts this. The two-photon absorption coefficient is about 3 × 10^−13^ m/W [94].

Due to interacting multiphoton ionization and/or tunneling ionization mechanisms, laser-induced high-field ionization has the potential to excite electrons in the conduction band [39]. Michele et al. [95] studied the interaction between an ultraviolet femtosecond laser and silica, and the results showed that the inter-band absorption of an ultraviolet femtosecond laser was acceptably under the multiphoton ionization limit. Yu and Nogami [96] reported that when a femtosecond laser interacted with glass, two-photon excitation was generated in SiO_2_ glass, while three-photon excitation was generated in ZnO-SiO_2_ glass. Lancry et al. [97] confirmed that the femtosecond laser processing of silica glass was triggered by Zener tunnel ionization and modified by subsequent multiphoton absorption and cascade shock ionization. Additionally, Rublack et al. [98] investigated the femtosecond laser ablation of silicon dioxide on silicon and found that the threshold of the selective ablation process was almost independent of the linear absorption coefficient of silicon but significantly decreased with the shortening of the pulse duration in the case of femtosecond laser processing.

### 2.2. Energy Transformation

**Figure 5:**
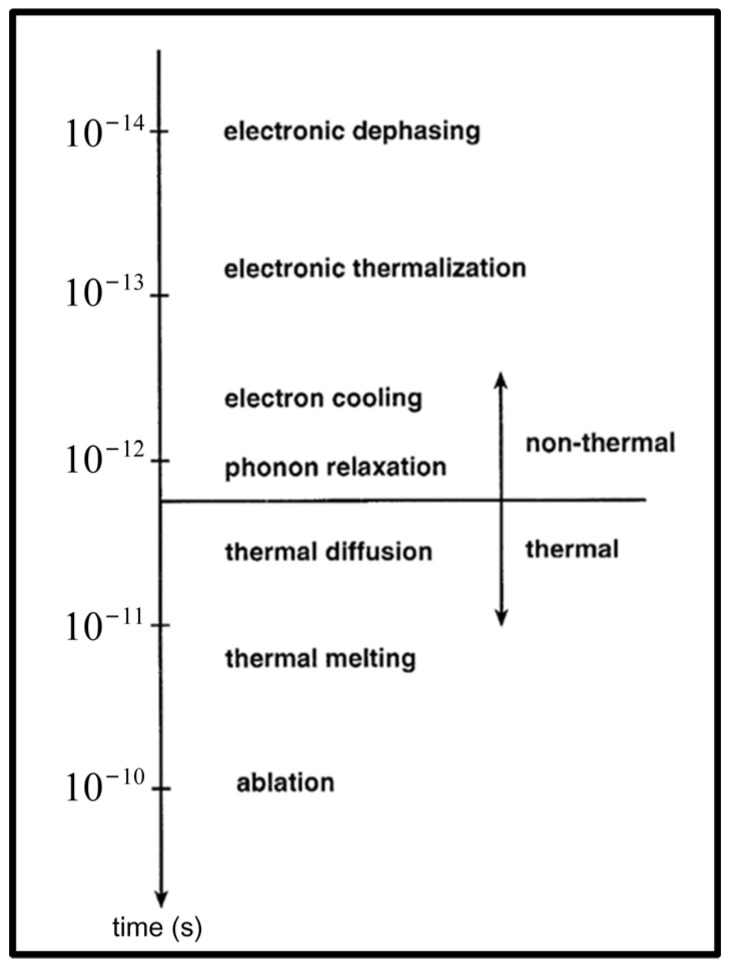
Time scale of the various secondary processes [79].

In ultrafast laser processing, the key to energy transfer and transformation is the interaction between electrons, ions, and photons in laser-induced plasma, as well as the interaction with continuum materials [99]. When the materials absorb a large amount of laser energy, high-temperature and high-pressure plasma will form, resulting in a series of complex secondary processes. A time scale of the physical process was proposed to describe the energy relaxation processes involved in femtosecond laser processing, as shown in Figure 5 [79]. In the non-thermal stage of the interaction between an ultrafast laser and a material, there are electronic dephasing (~10^−14^ s), electronic thermalization (~10^−13^ s), electron cooling, and phonon relaxation processes (~10^−12^ s). However, in the thermal stage, there are thermal diffusion, thermal melting (~10^−11^ s), and final ablation (~10^−10^ s) processes.

**Figure 6:**
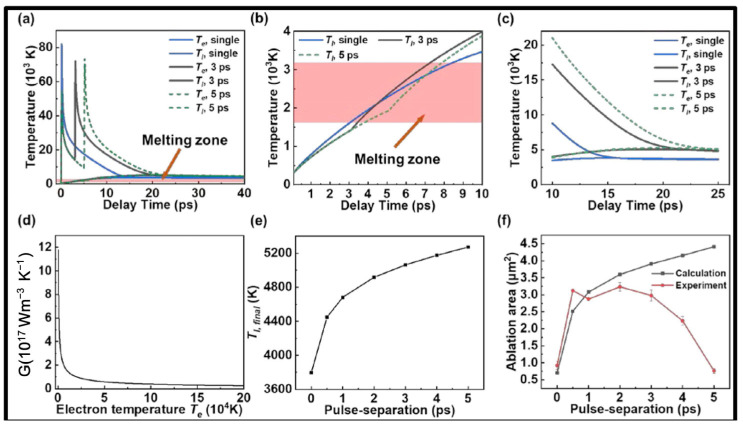
(**a**) Electron and lattice temperature evolution, (**b**) melted zone, (**c**) thermalization time, (**d**) electron–phonon coupling factor, (**e**) final lattice temperature, and (**f**) ablation area at different pulse separations [101].

Milosavljevic et al. [100] analyzed the interactions between a femtosecond laser and Nimonic 263 superalloy and reported that clear edges and the absence of a melted phase demonstrated direct solid–vapor conversion. Chen et al. [101] confirmed that the energy deposition from the electron system to the lattice system increased, and the ablation area also increased when using a double-pulse femtosecond laser for a nickel-based superalloy. Using the improved two-temperature model, the variation laws of electron temperature (T_e_) and lattice temperature (T_l_) with time were obtained, as shown in Figure 6 [101].

Boerner et al. [91] reported that an improved multi-rate equation model was introduced to study the electron density distribution and optical properties of the femtosecond laser processing of diamond and indicated that when using the improved multi-rate equation model, the damage and ablation thresholds could be calculated using electron density, absorbed energy density, or the electron temperature [102,103].

Additionally, Khmelnitski et al. [104] investigated the effect of electron dynamics on the graphitization of diamond when using a femtosecond laser and heavy ion irradiation and showed that the threshold energy required for the graphitization and destruction of a pre-damaged crystal when using a laser pulse was lower than that of an undamaged diamond. This was because the excess energy of the excited electronic subsystem in the lattice and the lifetime of these excited laser spots enhanced the graphitization of the initially damaged diamond sample compared to the undamaged diamond sample. Lu et al. [105] indicated that during high-power femtosecond laser processing, a strong plasma was generated, which would absorb the energy of the femtosecond laser, resulting in an irregular distribution of laser energy and the erosion of the micro-hole wall. Meanwhile, Zhang et al. [106] confirmed that when a femtosecond laser interacted with SiC, the main absorption mechanisms were multiphoton absorption and auger recombination. They also reported that thermal melting took place immediately after non-thermal melting when there was high flux in relation to the femtosecond laser and that the melt layer thickened logarithmically with increasing femtosecond laser flux.

## 3. Ultrafast Laser Processing of Brittle and Hard Materials

### 3.1. Nickel-Based Superalloys and Thermal Barrier Ceramics

The turbine blades of aircraft and power generation components have multi-layer configurations [107]. The typical three-layer configurations consist of a zirconia ceramic layer on the topcoat, an oxidation- and corrosion-resistant layer on the middle bond coat, and a superalloy as the substrate. These multi-layer-configuration components contain a mass of cooling holes [108]. Microsecond or nanosecond pulsed lasers have been used to drill cooling holes in aerospace components [109]. However, laser-induced defects, such as spatter, micro-cracks, heat-affected zones, recast layers, and delaminated ceramic thermal barrier coatings, are major issues involved in the drilling process when using microsecond or nanosecond lasers [110,111].

Recently, with ultra-short pulses (10^−15^ s), femtosecond lasers have been applied to manufacturing film-cooling holes on turbine blades and vanes [112,113,114].

Wei et al. [115] demonstrated that when compared with nanosecond laser drilling processes, femtosecond laser processes could significantly reduce the occurrence of recast layers on nickel-based single-crystal superalloys. Meanwhile, Barnett et al. [116] confirmed that the incidence of heat-affected zones and alterations to the sub-grain structure were not discovered in single-crystal superalloy after femtosecond laser machining. Additionally, Wang et al. [117] reported that in order to reduce the circularity error and taper of small holes, the machining parameters should be controlled to make the laser focus on the processing surface or slightly higher than the processing surface and confirmed that the morphology and inner wall quality of the holes machined using a femtosecond laser were much better than that of a traditional nanosecond laser; however, there were still thin recasting layers.

Recently, Zhang et al. [108,113] developed a femtosecond laser two-step method to drill holes in IN792 superalloy and confirmed that holes without heat-affected zones, oxidation zones, or recast layers were acquired through the use of the new method. Meanwhile, Dong and Li [114] verified that femtosecond laser drilling did not produce recast layers and micro-cracks on the cooling holes of a third-generation single-crystal superalloy, DD9. However, the femtosecond laser drilling of holes still had problems relating to roundness and taper deviation.

**Figure 7:**
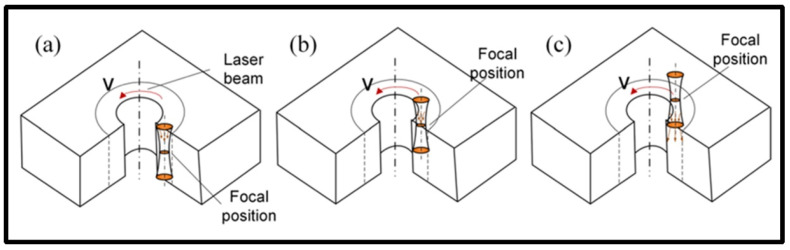
Schematic diagram of (**a**) negative focus position, (**b**) zero focus position, and (**c**) positive focus position [118].

In order to optimize the hole-making process when using a femtosecond laser, Sun et al. [118] developed a femtosecond laser drilling method to control the diameter, roundness taper, and material removal rate of K24 alloy holes, proving that the optimized laser drilling parameters improved the morphology and dimensional accuracy of the acquired micro-holes, as shown in Figure 7.

**Figure 8:**
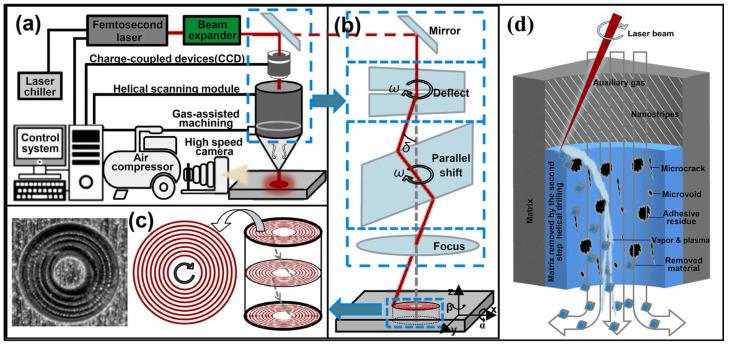
(**a**) Schematic diagram of femtosecond drilling system: (**b**) optical transmission principle of spiral scanning module, (**c**) scanning path, and (**d**) mechanism of two-step spiral drilling [120].

Yang et al. [119] indicated that a low scanning speed, small feed distance, medium scanning time, and high average laser energy were the basic parameters of K24 superalloy femtosecond laser drilling to produce high-quality micro-holes. Additionally, without changing the aperture of the entrance of the micro-holes, a low laser power could be used to repair the micro-holes obtained by the high laser energy, which could improve the appearance and roundness of the outlet hole and reduce the overall taper of the micro-holes when using the femtosecond laser process [105]. Meanwhile, Li et al. [120] developed a femtosecond laser two-step auger drilling method to achieve ultra-high-quality machining of film-cooling holes in a nickel-based single-crystal superalloy for turbine blades, as shown in Figure 8. Additionally, Xiao et al. [121] indicated that the stress distribution and stress axis direction of a Ni-based single-crystal superalloy were effectively changed when changing the femtosecond laser drilling direction, which significantly affected the anisotropic creep behavior of the single-crystal superalloy with film-cooling holes as well as the deformation mechanisms.

Recently, Wei et al. [122] indicated that the two material removal mechanisms relating to an Inconel 738 substrate when using the femtosecond laser process were normal vaporization on the upper surface and phase explosion on the lower surface of the drilling holes. However, Li et al. [123] claimed that the upper surface of the micro-hole wall was smooth and the bottom surface of the micro-hole had a capillary structure after the femtosecond laser drilling of a nickel-based superalloy due to the re-ablation of laser-induced plasma and the uneven pressure gradient in the inner area of the micro-hole. Yu et al. [124,125] reported that in the ablation zone of the thermal barrier coating of a DD6 single-crystal superalloy when using femtosecond laser shock drilling, the main ablation mechanism was phase explosion accompanied by droplets, gas phase molecules, and small molecule clusters. However, in the laser-induced zone, the main ablation mechanism was vaporization, and moderate melting was also common. Additionally, Qiu et al. [90] developed an innovative method for machining high-precision micro-holes with small tapers using a femtosecond laser, which could be used to machine high-quality micro-holes in a wide range of industrial applications.

### 3.2. Diamond

Diamond has excellent properties, such as high hardness, thermal conductivity, brilliance, and reflectivity, making diamond an important material in industries such as those that involve cutting tools, semiconductors, and electronics [126]. Traditional diamond processing methods include electrical discharge [127,128], mechanical grinding [129], and waterjet processing [130]. However, there are still problems involving low processing precision, complex processes, low efficiency, and environmental pollution in diamond processing when using traditional methods. Recently, high-precision femtosecond laser processes have been widely used for diamond processing [131,132,133]. Compared with the relatively long pulse durations of nanosecond lasers [134,135] and picosecond lasers [136,137], the pulse duration of a femtosecond laser is 10^−15^ s, shorter than the time (10^−10^–10^−12^ s) of the electron–lattice relaxation process. Thus, the cracks, heat-affected zone, and graphitization of machined diamond surfaces could be restrained.

**Figure 9:**
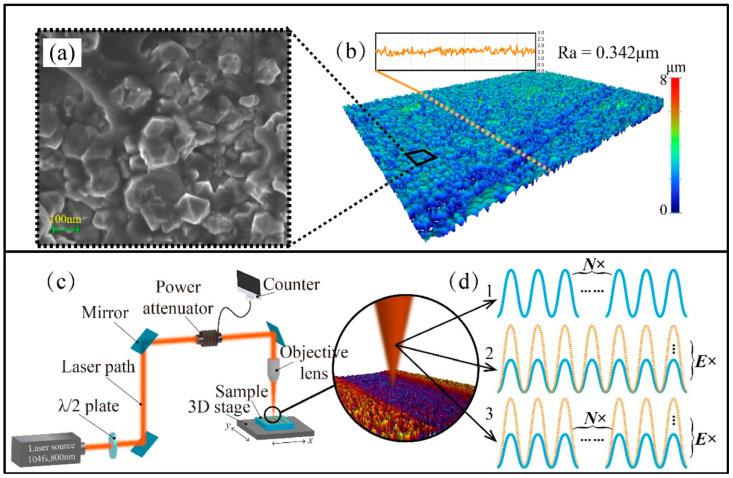
Materials and schemes diagram of femtosecond laser processing: (**a**) SEM photograph of diamond; (**b**) three-dimensional morphology of the diamond; (**c**) femtosecond laser processing system; and (**d**) three experimental schemes: change only the pulse number, N, only the single pulse laser energy, E, and simultaneously change N and E [138].

Wei et al. [138] reported that there were two mechanisms involved in the ablation of diamond when using a femtosecond laser. On the one hand, in femtosecond laser processing, the diamond surface would undergo material melting and re-solidification at a high scanning velocity or low single-pulse laser energy. On the other hand, when the scanning velocity decreased or the pulse laser energy increased, the ablation of the diamond coating became a three-stage mechanism of melting, graphitization, and evaporation (Figure 9).

Khomich et al. [139] indicated that three femtosecond graphitization regimes for diamond were realized when using different femtosecond laser irradiation energies. Firstly, when the energy approached the threshold, the graphitization rate was dependent on the penetration of the light field into the crystal. Secondly, with increased irradiation energy, the penetration depth of the graphitization front suddenly became saturated. Finally, when the femtosecond laser energy density surpassed a certain threshold, bulk graphitization was achieved in the crystal volume of the diamond. Additionally, Bakhtiar et al. [140] confirmed that when the fluence of femtosecond laser irradiation was raised to 3.9 J/cm^2^, the diamond’s tetrahedral sp^3^ phase converted to the sp^2^ aromatic phase with a high degree of crystallinity without the formation of a heat-affected zone or thermal cracking. However, when the fluence was above 3.9 J/cm^2^, the size of the planar crystalline domains in the femtosecond photo-irradiated regions was significantly reduced; however, the sp^2^ aromatic clustering was preserved. Kononenko et al. [141] reported that the laser process occurred across three different states depending on the femtosecond laser energy. In the first state, highly oriented graphite was achieved at a minimum laser flux of 3–4 J/cm^2^. In the second state, with the development of ablation, the graphitized material became amorphous when applying a femtosecond laser flux of about 4–20 J/cm^2^. Finally, when the flux of the femtosecond laser was greater than 20 J/cm^2^, the thickness of the graphitized layer significantly increased to about 1 µm. The graphitized layer could be considered to block graphitization.

Recently, Rossi et al. [142] analyzed the phase transition, structural defects, and stress development of a femtosecond-laser-machined diamond surface and buried zone and concluded that the conduction of the electrodes was carried out through both a conductive graphite phase and a disordered diamond path and that the performance of the detector was mainly related to the charge transport inside the unprocessed diamond. Lin et al. [143] confirmed that optical gratings and wires on the surface and inside of diamond sheets were successfully fabricated using a femtosecond laser, although only part of the materials in the irradiation zone changed from a single crystalline phase to an amorphous phase. Meanwhile, Ashikkalieva et al. [144] reported that the internal nanostructures and electrical properties of graphitized lines had been successfully produced in a single-crystal diamond through the use of femtosecond laser processing. Rotating the laser beam inside the diamond crystal resulted in a considerable change in the arrangement of the laser-induced sp^2^ nanosheets along the planar orientation of the diamond crystal. However, the improved femtosecond laser pulse width increased the conductivity of the wire and the thickness of sp^2^ inclusion in the studied range. Additionally, Mastellone et al. [145] reported that a vertical microstructure within diamond blocks was achieved via laser-induced phase transitions from diamond to graphite using a femtosecond laser, demonstrating the feasibility of graphite microwires embedded in diamond because of their robustness in terms of high-temperature applications, such as their use in thermionic emitters.

### 3.3. SiO_2_ and Silica Glass

With favorable physical and chemical properties, silicon dioxide (SiO_2_) is widely used in aerospace, optics, electronics, and other fields [146,147,148]. However, SiO_2_ is difficult to machine due to its high hardness [149].

**Figure 10:**
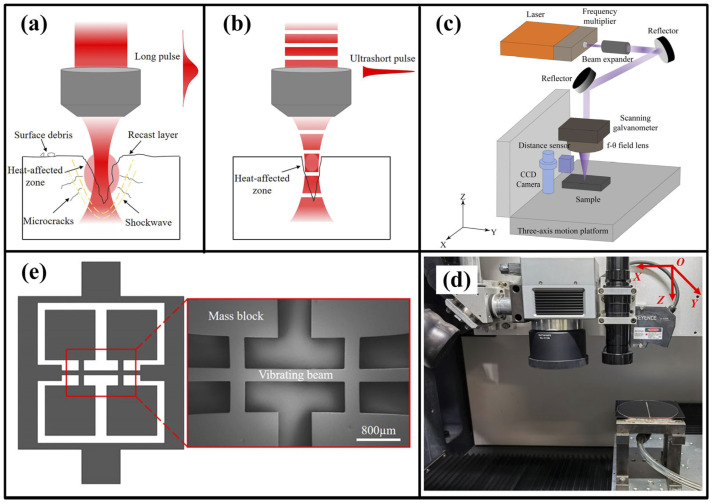
Schematic diagram of (**a**) long pulsed laser, (**b**) ultrashort-pulsed laser, (**c**) femtosecond laser processing system, (**d**) photograph of the system, and (**e**) resonance microstructure [154].

Recently, when compared with traditional methods, the femtosecond laser process has been regarded as a crucial method for micromachining SiO_2_ due to its significant advantages, such as its ultrashort pulse duration, high precision, and negligible damage to material surfaces [150,151,152,153]. Jian et al. [154] reported that a breakthrough in the precision of femtosecond laser processing of silica manufacturing had been achieved and that the efficiency of femtosecond laser processing had also been greatly improved (Figure 10).

**Figure 11:**
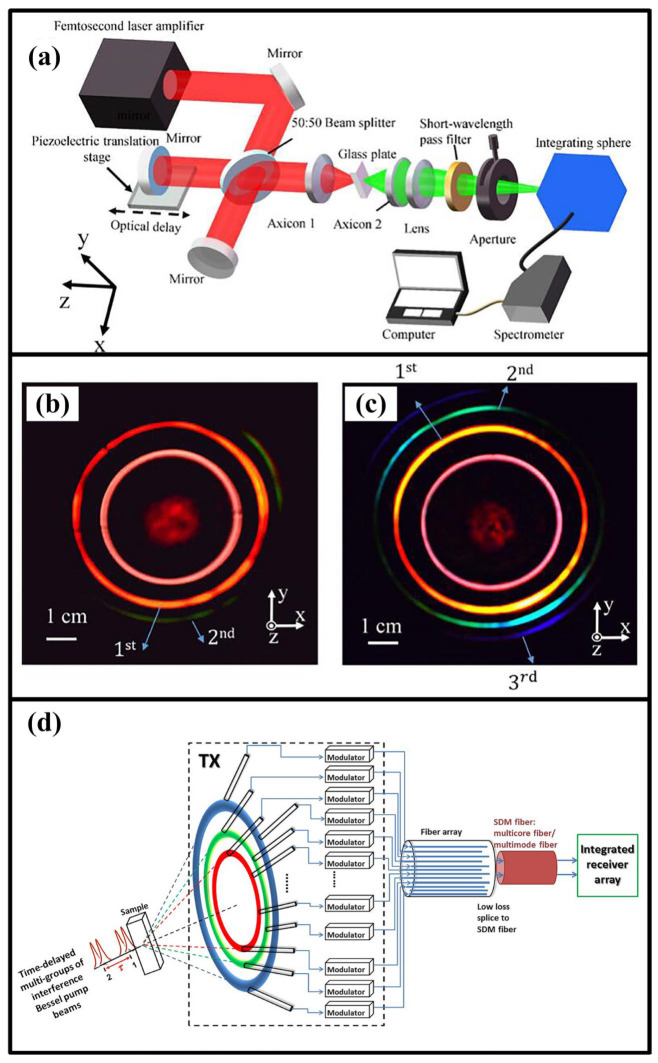
(**a**) Experimental setup used to generate the multi-wavelength ultrashort annular beams using double femtosecond Bessel laser beams; beam cross-sectional images after the silica glass when single (**b**) and double (**c**) femtosecond Bessel laser beam(s) were used, respectively. The single pulse energies used in (**b**,**c**) are 0.4 mJ and 0.2 mJ, respectively. (**d**) Schematic diagram of a potential application of multidimensional multiplexing optical communication system [155].

Liu et al. [155] prepared multi-wavelength toroidal pulses in silica glass by using a double-interference femtosecond Bessel laser beam and confirmed that the efficiency of the toroidal pulses could be greatly improved when compared with that of a single-interference femtosecond Bessel laser beam (Figure 11).

**Figure 12:**
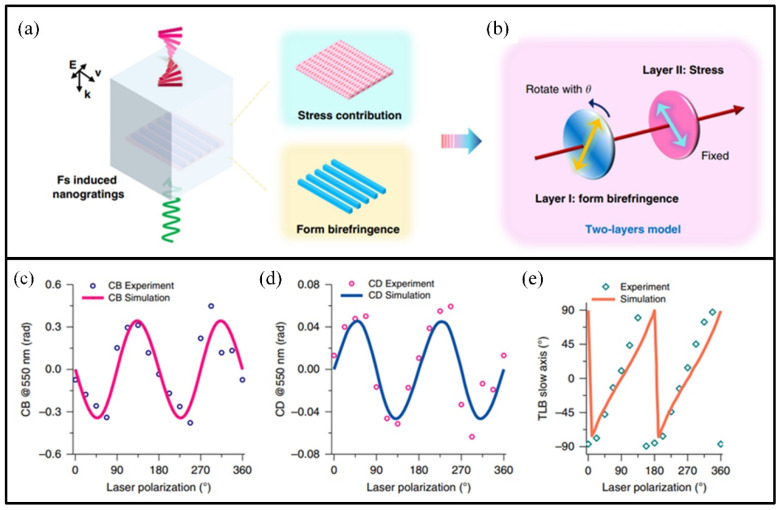
Two-layer model and simulation results: (**a**) schematic diagram of the two contributions, (**b**) two-layer model consists of two linear retarders; (**c**,**d**) the evolution of laser radiation is simulated based on laser polarization and compared with experimental results; (**e**) calculations and measurements results [157].

Yang et al. [156] reported that a femtosecond laser could be used to enhance the strong light emission of SiO_2_ crystal materials by combining nanoparticle-enhanced laser-induced breakdown spectroscopy with double-pulse laser-induced breakdown spectroscopy. Lu et al. [157] also proposed a femtosecond laser direct writing phenomenon model for silica glass based on a two-layer phase shifter, which could explain the optical chirality of laser-induced achiral materials (Figure 12).

**Figure 13:**
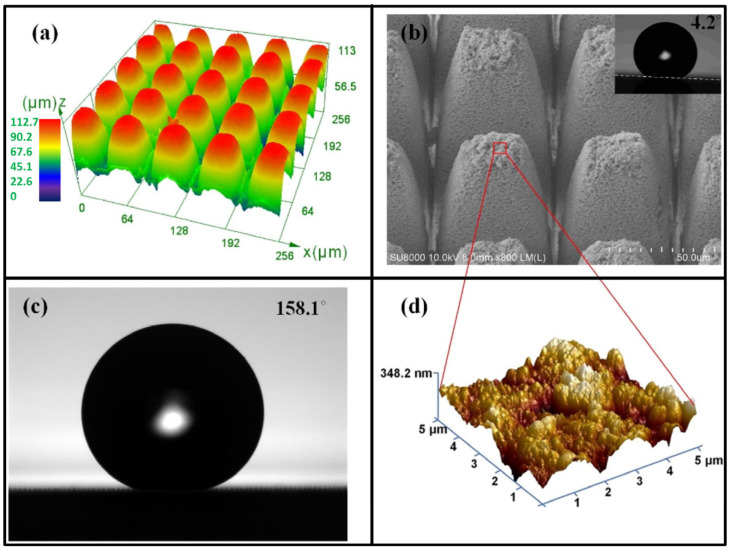
Surface morphology and water contact angle of superhydrophobic glass obtained using the optimal parameters: (**a**) laser scanning confocal microscope, (**b**) microstructure and sliding angle, (**c**) water contact angle, and (**d**) atomic force microscopy [160].

Additionally, the damage to the structure of silica glass when subjected to femtosecond laser processes has been widely investigated with consideration of the optical characteristics required of silica in the field of electronics [158,159]. Recently, Liao et al. [160] reported that an effective coalescing of a silica glass microchannel structure and drag-reducing functional microstructure was obtained through the use of femtosecond laser technology (Figure 13).

### 3.4. SiC and Composites

Silicon carbide (SiC) is widely used in high-temperature, high-speed, and high-voltage electronic devices because of its excellent properties, such as its high thermal conductivity, high thermal stability, wide bandgap, and high saturation drift speed [161,162,163,164]. However, due to its excellent chemical stability and high hardness and brittleness, silicon carbide is difficult to efficiently machine at high quality when using traditional methods such as chemical etching, tool machining, and photolithography processing [165,166,167].

**Figure 14:**
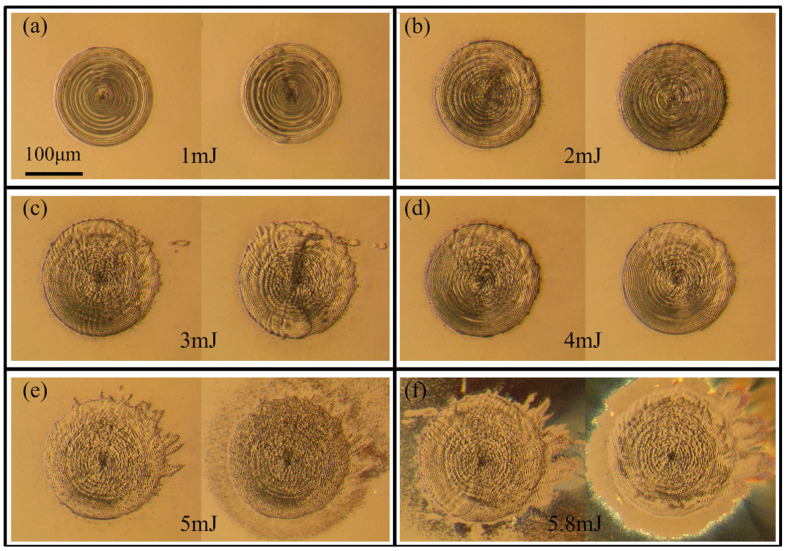
The microstructure of silica with different laser pulse energies: (**a**) 1 mJ, (**b**) 2 mJ, (**c**) 3 mJ, (**d**) 4 mJ, (**e**) 5 mJ, and (**f**) 5.8 mJ [172].

Recently, femtosecond laser machining has been widely applied in the precision manufacturing of silicon carbide due to its advantages in terms of high precision, low roughness, and lack of heat-affected zone, resulting in consistent high quality and efficiency [168,169,170]. Wu et al. [171] demonstrated that the ablation threshold was closely related to the number of femtosecond laser pulses and the processing medium used. Additionally, surface roughness and oxidation were reduced with the help of the liquid machining media. Thus, specific surface requirements for the manufacturing of SiC devices were achieved when using the liquid machining media. Zhang et al. [172] indicated that a concentric ring structure was acquired by focusing a single femtosecond pulse on the surface of molten silica, which indicated that the concentric ring structure had good application prospects in the preparation of isotropic structurally colored surfaces (Figure 14).

**Figure 15:**
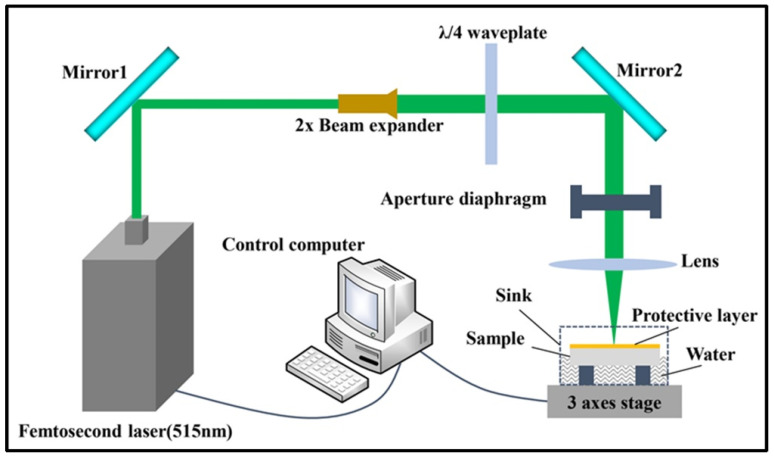
Experimental setup of the femtosecond laser drilling system [173].

Additionally, Liu et al. [173], with the help of a protective layer, applied femtosecond lasers to obtain high-quality micro-holes without micro-cracks at the inlet and outlet. They also had smooth inner walls, which are of great significance for the development of SiC-based electronic devices and the high-quality precision machining of other brittle, hard, and unworkable materials (Figure 15).

**Figure 16:**
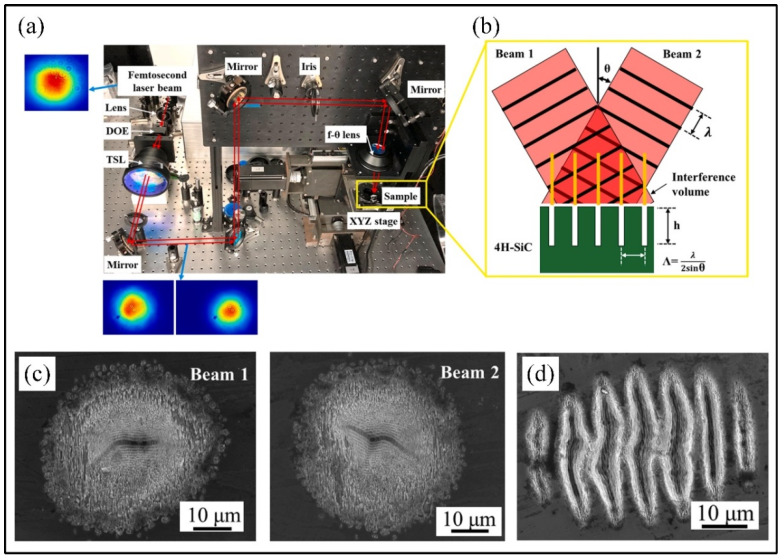
(**a**) The ultrashort-pulse direct laser interferogram experimental system, (**b**) the schematic of the process, (**c**) microstructure produced by two laser beams without interference, and (**d**) microstructure produced using two-beam direct laser interferogram [174].

Liu et al. [174] reported that the surface microstructure of 4H-SiC was successfully generated using an ultrashort-pulse direct laser interferogram and single Gaussian beam process (Figure 16) and confirmed that the Raman peak of crystal SiC after 4H-SiC irradiation using an ultrashort-pulse direct laser interferogram was greater than that of single-beam irradiation due to the fact that ultrashort-pulse direct laser interferogram could use the superposition of two femtosecond laser beams to realign the laser energy distribution.

**Figure 17:**
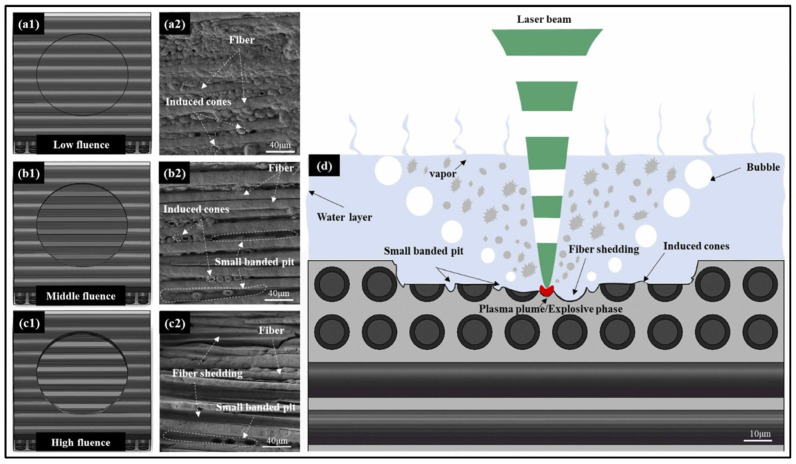
The morphology with different parameters: (**a1**,**a2**) low energy (**b1**,**b2**) middle energy, (**c1**,**c2**) high energy, and (**d**) removal mechanism [175].

Silicon carbide ceramic matrix composites are widely applied in aerospace and energy fields because of their advantages in terms of their high hardness, high strength, and low density. Wei et al. [175] developed a new method of underwater femtosecond laser ablation for SiC/SiC composites and revealed the removal mechanism involving SiC/SiC composites, providing new research ideas for surface oxidation resistance processing, as shown in Figure 17.

**Figure 18:**
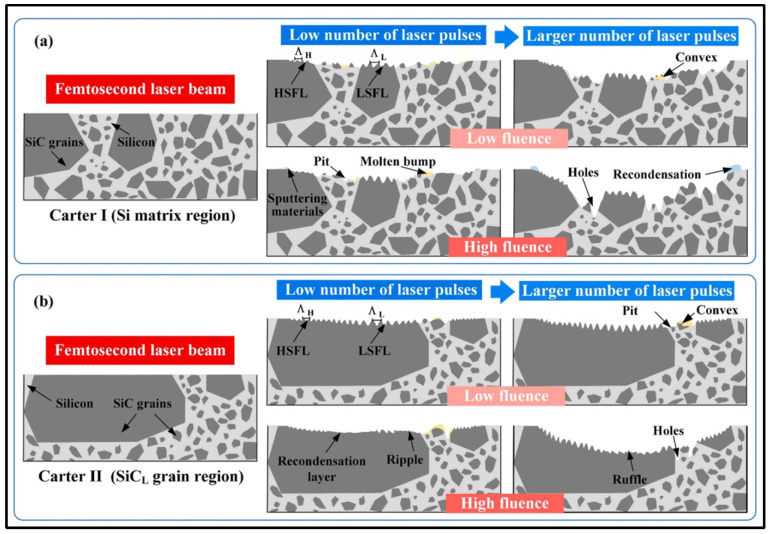
Schematic diagram of the ablation mechanism: (**a**) Si matrix zone and (**b**) SiC_L_ grain zone [176].

Recently, by analyzing the surface microstructure and phase of the ablation pit of reactor-bonded silicon carbide composites, a preliminary model of the ablation pit removal mechanisms when using femtosecond laser irradiation was established, as shown in Figure 18 [176].

## 4. Precise Manufacturing of Ultrafast Laser Processing

### 4.1. Processing Quality Control

**Figure 19:**
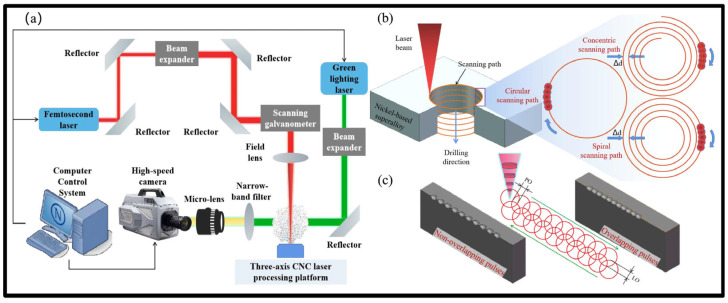
Schematic diagram of (**a**) femtosecond laser processing system, (**b**) laser scanning paths, and (**c**) calculation of overlap rate [179].

The coupling of an ultrafast laser processing mechanism model and a data model is a major issue to solve in terms of precise manufacturing [177,178]. The mechanism model of ultrafast laser processing contains prior knowledge and algorithms used in the fields of laser energy absorption, transfer and transformation, phase transformation and ablation, etc. This model has the characteristics of high theoretical accuracy and wide universality. However, it is usually reasonably simplified in terms of solutions. It is prone to deviation influenced by the difference between the practical and theoretical aspects of laser processing. Based on the data paradigm, the data model can be used to better solve the problems involved in high-order and nonlinear complex systems. However, due to natural uncertainties, the coupling degree between the data model and traditional mechanism model-driven processing technology modeling is weak, making it difficult to realize regularity and predictability in terms of data analysis. Thus, clarifying the mechanisms involved in the fusion and mutual relationship between the mechanism model and the data model is a key issue in process modeling. Dong et al. [179] demonstrated that in the range of femtosecond laser pulse overlap, the micro-hole size remained relatively stable. The taper was the smallest when the pulse overlap rate was 92.5%, and the lower pulse overlap rate improved the quality of the micro-hole wall and reduced the appearance of recast layers and micro-cracks (Figure 19).

Recently, Zhang et al. [180] indicated that the femtosecond laser ablation efficiency of a SiC ceramic matrix composite increased with femtosecond laser pulse energy, which was positively correlated with groove width, depth, heat-affected zone width, and side slope angle. Meanwhile, by controlling the energy of an 800 nm femtosecond laser and applying isopropyl alcohol, Zhang et al. [181] controlled a sub-diffractive-limited lithography with a microstructure of about 30 nm acquired on the surface of a diamond film and diamond gratings within a period of 200 nm were also obtained. Additionally, Zhang et al. [182] investigated the femtosecond laser processing of single-crystal silicon carbide and verified that the numerical aperture had significant effects on the depth, width, width of the heat-affected zone, and the slope angle of the side wall.

### 4.2. Precise Manufacturing System

**Figure 20:**
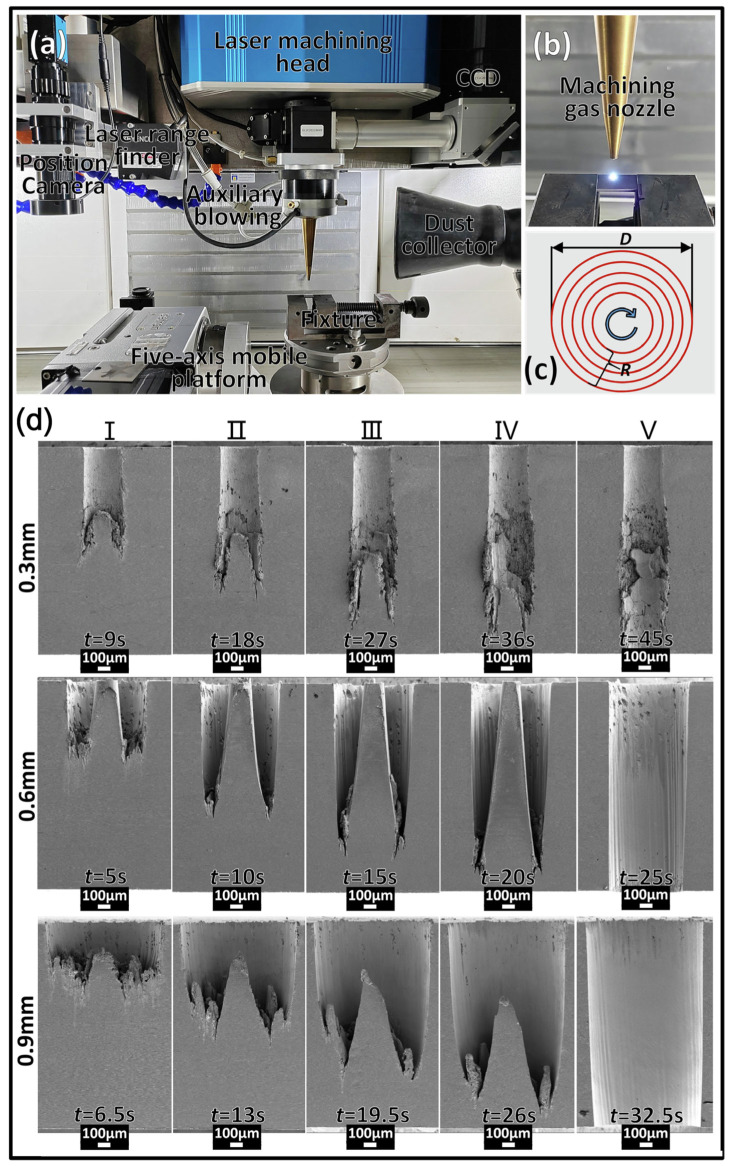
(**a**) A femtosecond helical drilling system, (**b**) machining area, (**c**) helical scanning trajectory, and (**d**) microstructure profiles of hole walls produced with different drilling intervals [191].

**Figure 21:**
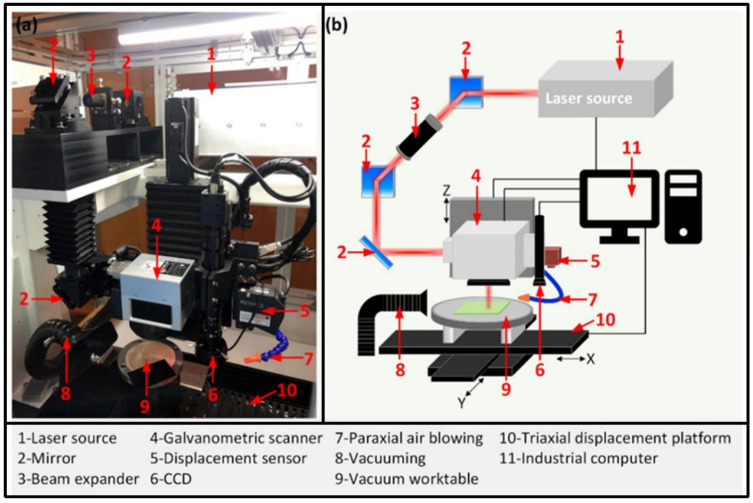
(**a**) Picture and (**b**) schematic of the laser workstation [192].

Ultrafast laser processing has become the most promising new technology for high-precision etching, drilling, cutting, and microstructural preparation of virtually all types of materials [183,184,185,186]. However, precise ultrafast laser processing involves not only motion axis control but also laser parameter control, optical path direction control, and galvanometer and objective control with relatively high real-time requirements [187,188,189,190]. Recently, Li et al. [191] reported that with an increased femtosecond laser scanning diameter, the taper of the micro-hole also increased, and the processing efficiency enhanced at first and then reduced. Additionally, the surface of the micro-hole wall changed from micro-holes, micro-cracks, and oxidized rubber residues to regular nanofringes, as shown in Figure 20. Meanwhile, Wang et al. [192] presented that the modification threshold and microstructure transformation threshold of SiC processed using a femtosecond laser were 2.35 J/cm^2^ and 4.97 J/cm^2^. When the effective pulse number of a femtosecond laser reached 720, the ablation threshold of SiC decreased to 0.70 J/cm^2^ due to the energy accumulation effect of a femtosecond laser (Figure 21).

**Figure 22:**
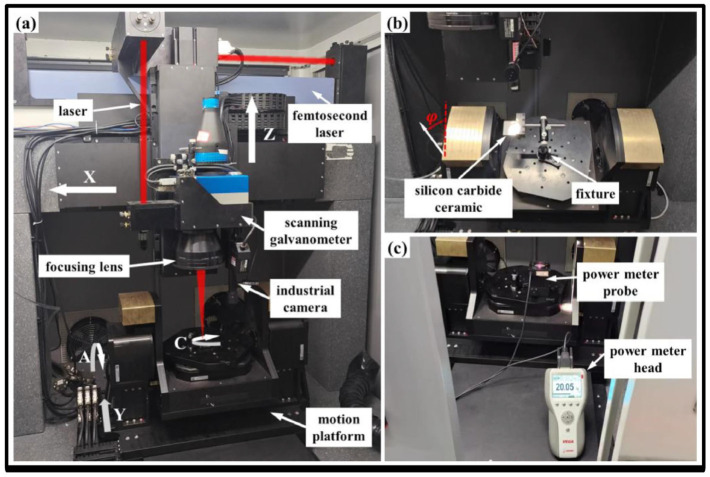
Diagram of (**a**) femtosecond five-axis micromachining system, (**b**) variable angle, and (**c**) laser energy measurement [194].

**Figure 23:**
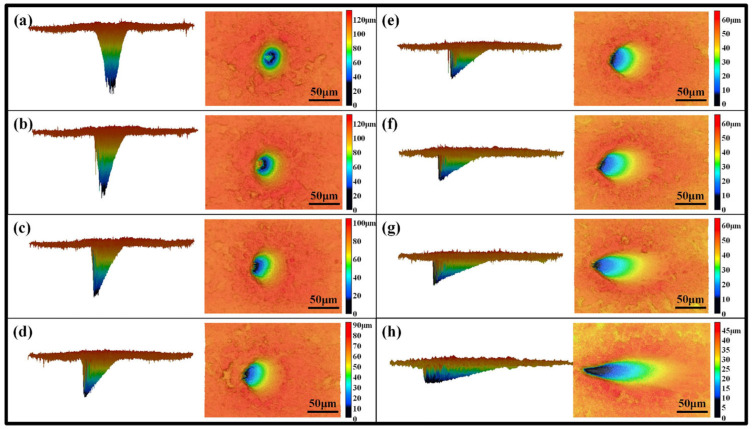
The ablation crater microstructure with various laser incident angles: (**a**) 0°, (**b**) 10°, (**c**) 20°, (**d**) 30°, (**e**) 40°, (**f**) 50°, (**g**) 60°, and (**h**) 70° [194].

The quality of precise femtosecond laser manufacturing is a challenge because femtosecond laser processing involves many parameters, and the coupling between the parameters is also complex. However, femtosecond lasers are one of the most effective methods for fabricating silicon carbide with high efficiency and high quality [193]. Additionally, Chen et al. [194] developed a five-axis infrared femtosecond laser machining system and analyzed the femtosecond laser micromachining of SiC ceramics with different process parameters. The results showed that with the increase in the incidence angle of the femtosecond laser, the ablation threshold of SiC ceramics first increased, then decreased, and finally increased, as shown in Figure 22 and Figure 23.

### 4.3. Artificial Intelligence-Assisted Manufacturing

**Figure 24:**
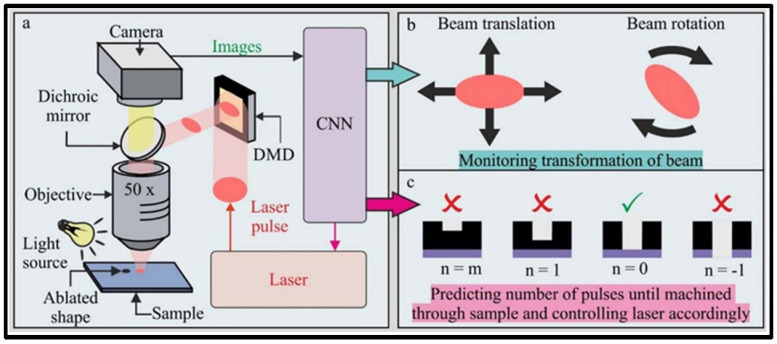
Schematic diagram of (**a**) real-time closed-loop feedback in femtosecond laser micromachining, (**b**) detecting the transformation of the laser beam, and (**c**) forecasting the remaining number of pulses [198].

Recently, the efficiency and quality of manufacturing have been greatly improved due to the rapid development of artificial intelligence [195,196,197]. Xie et al. [198] reported that by visually monitoring the materials to be processed during femtosecond laser micromachining, a neural network for system monitoring was developed. They confirmed that unintentional laser beam translation and rotation could be simultaneously detected during femtosecond laser micromachining, thus realizing the feasibility of simultaneously identifying multiple femtosecond laser micromachining parameters and greatly improving the processing quality of femtosecond laser micromachining (Figure 24).

**Figure 25:**
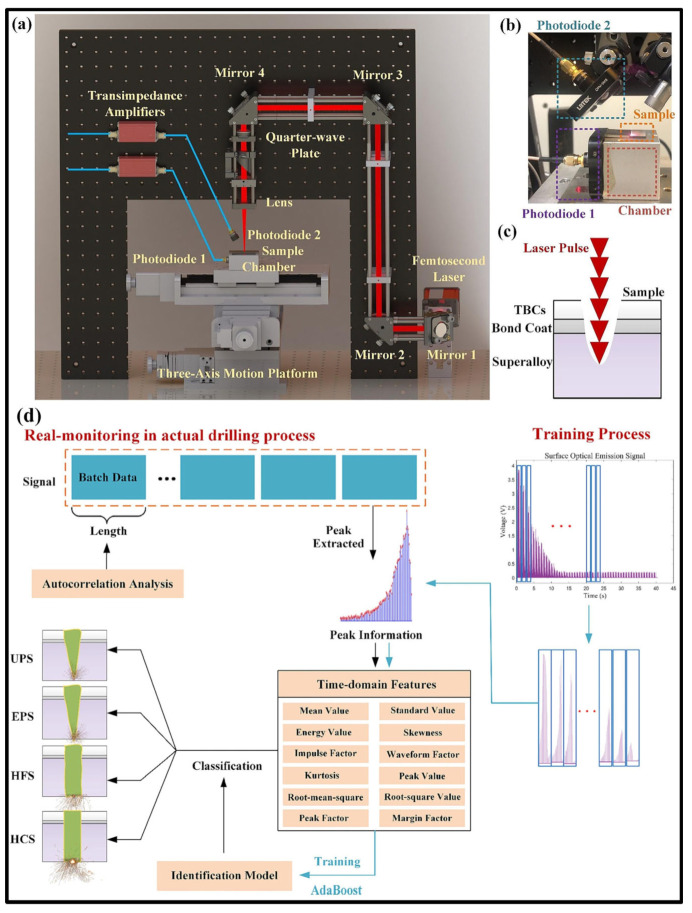
Schematic diagram: (**a**) femtosecond laser drilling system with monitoring platform, (**b**) photodiode and material positions, (**c**) femtosecond laser percussion drilling, and (**d**) real monitoring process [74].

Meanwhile, Sun et al. [74] designed a data-driven femtosecond laser micromachining method and established a classification model of different film layer cooling holes for the femtosecond laser drilling stage. A set of real-time monitoring and process control models for the femtosecond laser drilling process was proposed, providing new ideas and a scheme for solving the problem of excessive ablation during femtosecond laser micromachining (Figure 25).

**Figure 26:**
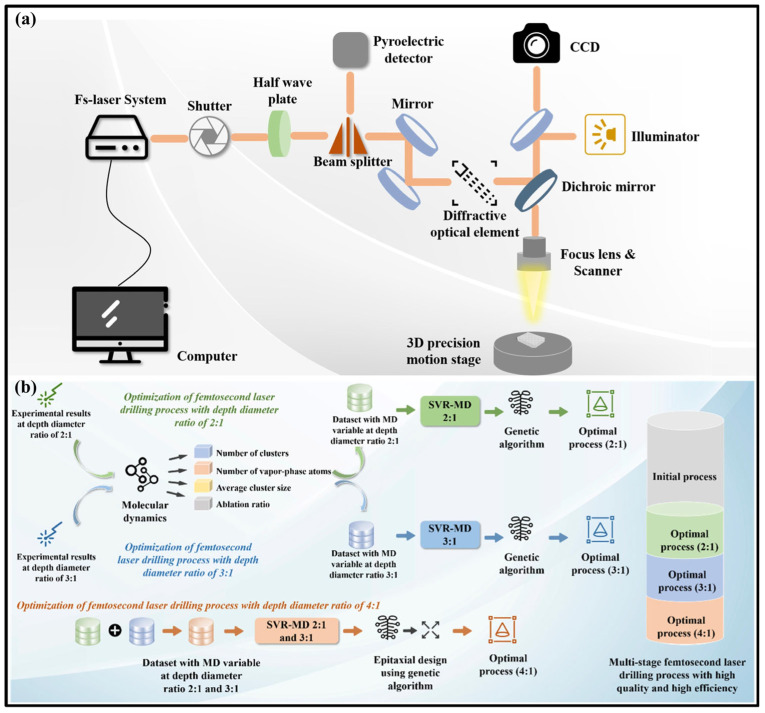
Schematic diagram of (**a**) femtosecond laser percussion micromachining system and (**b**) four-stage percussion laser drilling process [199].

Additionally, Wang et al. [199] confirmed that a process optimization model and scheme combining molecular dynamics simulation, machine learning, and a high-throughput optimization algorithm for femtosecond laser drilling were established, proving that machine learning could quickly and accurately establish a regression model between femtosecond laser parameters and target machining performance. It was determined that the high-throughput optimization algorithm was responsible for determining the optimal femtosecond laser machining process in terms of process control, achieving high-quality and high-efficiency femtosecond laser micromachining (Figure 26).

**Figure 27:**
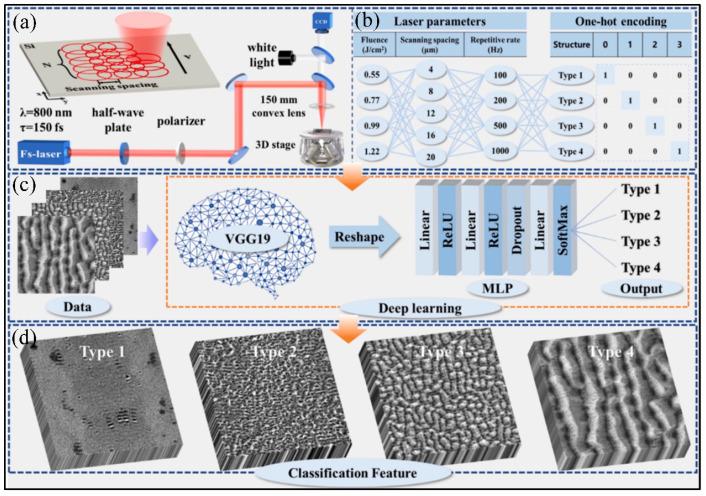
Schematic diagram of femtosecond laser processing and deep learning: (**a**) femtosecond laser micromachining, (**b**) encoding process of microstructure types with different parameters, (**c**) deep learning flow diagram, and (**d**) typical microstructure [200].

Recently, Ye et al. [200] also invented a structure optimization method based on data-driven deep learning combined with a laser energy deposition model to control the microstructure and properties of materials produced using femtosecond laser processing (Figure 27).

## 5. Conclusions and Perspective

Ultrafast laser processing has become an effective method for the high-quality processing of brittle and hard materials, such as nickel-based superalloys, thermal barrier ceramics, diamond, SiO_2_, silica glass, SiC, and composites, which are widely used and developed for use in optical and aerospace applications, semiconductors, and so on. The absorption and transfer of femtosecond laser energy are two key factors in the interaction between femtosecond pulsed lasers and hard and brittle materials. The efficiency and quality of ultrafast laser manufacturing has been greatly improved by the rapid development of artificial intelligence. However, challenges, promising approaches, risks and needed considerations relating to the femtosecond laser processing of brittle and hard materials still exist:

(1) The nonlinear laser energy absorption mechanisms of brittle and hard materials with different physical properties are still unclear. An in situ monitoring system with a high spatiotemporal resolution could be established by integrating spectral detection and three-dimensional ultrafast continuous imaging into an SEM system in order to analyze the evolution of laser-induced plasma and the absorption process of the femtosecond laser energy.
(2) The coupling mechanism of laser energy transfer and the transformation of brittle and hard materials is still uncertain. Based on time-dependent density functional theory, molecular dynamics theory, and continuum theory, a multi-scale theoretical model of femtosecond laser machining could be established to carry out dynamic simulations of femtosecond laser processing. Meanwhile, combined with the above-mentioned in situ monitoring system, the evolution process of laser-induced plasma, three-dimensional morphology, and material state changes could be obtained to examine the non-equilibrium energy transfer and transformation mechanisms involved in the femtosecond laser cross-scale processing of the photon–electron–ion continuum.
(3) The processing dimensional accuracy and shape accuracy of brittle and hard materials when using a femtosecond laser still need to be improved. The flexibility and stability control of laser energy parameters, such as pulse energy, pulse width, and focus position, is key to realizing high-precision femtosecond laser processing. Coordinated regulation based on the time, space, and frequency domains of femtosecond laser processing is a promising method for controlling laser energy parameters.
(4) Ensuring quality consistency in the large-format femtosecond laser processing of brittle and hard materials is also a challenge. Real-time adjustments of a laser beam’s motion parameters, such as incident angle, trajectory, attitude, speed, etc., are essential to ensure consistent quality. With the integration of high-precision motion platforms, process monitoring systems, and interpolation algorithms relating to geometry, pose, and velocity, the motion parameters of the laser beam could be effectively controlled to acquire accurate processing paths, speeds, and attitudes relating to femtosecond laser processing and achieve high consistency.
(5) Artificial intelligence-assisted ultrafast laser manufacturing needs to be considered. Artificial intelligence technologies, such as neural networks, machine learning, deep learning, and reinforcement learning, have good application potential in terms of establishing the relationship between process parameters and quality, as well as the intelligent planning of process parameters and the robust online optimization of process parameters in the ultrafast laser processing of brittle and hard materials. Artificial intelligence-assisted manufacturing would save a lot of time and money, be beneficial to the final properties, and improve processing efficiency and quality.
